# Central and peripheral α‐synuclein in Parkinson disease detected by seed amplification assay

**DOI:** 10.1002/acn3.51753

**Published:** 2023-03-27

**Authors:** Lana M. Chahine, Thomas G. Beach, Charles H. Adler, Monica Hepker, Anumantha Kanthasamy, Scott Appel, Sandra Pritzkow, Michelle Pinho, Sherri Mosovsky, Geidy E. Serrano, Christopher Coffey, Michael C. Brumm, Luis M. A. Oliveira, Jamie Eberling, Brit Mollenhauer, For the Systemic Synuclein Sampling Study, Kuldip Dave, Danna Jennings, John Seibyl, Vanessa Arnedo, Lindsey Riley, Carly Linder, Tatiana Foroud, Katherine Kopil, David Russell, Penelope Hogarth, Rizwan Akhtar, Amy Amara, Connie Marras, Naomi Visanji, David P. Breen, John Crary, Charles White, David Munoz, Chelsea Caspell‐Garcia

**Affiliations:** ^1^ Department of Neurology University of Pittsburgh Pittsburgh Pennsylvania USA; ^2^ Banner Sun Health Research Institute Sun City Arizona USA; ^3^ Department of Neurology Mayo Clinic College of Medicine Scottsdale Arizona USA; ^4^ Biomedical Sciences Iowa State University Ames Iowa USA; ^5^ Center for Brain Science and Neurodegenerative Diseases, Department of Physiology and Pharmacology University of Georgia Athens Georgia USA; ^6^ Biostatistics Analysis Center University of Pennsylvania Philadelphia Pennsylvania USA; ^7^ Department of Neurology University of Texas, McGovern Medical School Houston Texas USA; ^8^ Department of Biostatistics University of Iowa College of Public Health Iowa City Iowa USA; ^9^ The Michael J. Fox Foundation for Parkinson's Research New York New York USA; ^10^ Center of Parkinsonism and Movement Disorders, Department of Neurology Paracelsus‐Elena Klinik Kassel and University Medical Center Göttingen Göttingen Germany

## Abstract

**Objectives:**

Detection of α‐synuclein aggregates by seed amplification is a promising Parkinson disease biomarker assay. Understanding intraindividual relationships of α‐synuclein measures could inform optimal biomarker development. The objectives were to test accuracy of α‐synuclein seed amplification assay in central (cerebrospinal fluid) and peripheral (submandibular gland) sources, compare to total α‐synuclein measures, and investigate within‐subject relationships.

**Methods:**

The Systemic Synuclein Sampling Study aimed to characterize α‐synuclein in multiple tissues and biofluids within Parkinson disease subjects (*n* = 59) and compared to healthy controls (*n* = 21). Motor and non‐motor measures and dopamine transporter scans were obtained. Four measures of α‐synuclein were compared: seed amplification assay in cerebrospinal fluid and formalin‐fixed paraffin‐embedded submandibular gland, total α‐synuclein quantified in biofluids using enzyme‐linked immunoassay, and aggregated α‐synuclein in submandibular gland detected by immunohistochemistry. Accuracy of seed amplification assay for Parkinson disease diagnosis was examined and within‐subject α‐synuclein measures were compared.

**Results:**

Sensitivity and specificity of α‐synuclein seed amplification assay for Parkinson disease diagnosis was 92.6% and 90.5% in cerebrospinal fluid, and 73.2% and 78.6% in submandibular gland, respectively. 25/38 (65.8%) Parkinson disease participants were positive for both cerebrospinal fluid and submandibular gland seed amplification assay. Comparing accuracy for Parkinson disease diagnosis of different α‐synuclein measures, cerebrospinal fluid seed amplification assay was the highest (Youden Index = 83.1%). 98.3% of all Parkinson disease cases had ≥1 measure of α‐synuclein positive.

**Interpretation:**

α‐synuclein seed amplification assay (cerebrospinal fluid>submandibular gland) had higher sensitivity and specificity compared to total α‐synuclein measures, and within‐subject relationships of central and peripheral α‐synuclein measures emerged.

## Introduction

α‐synuclein (αSyn) is the pathologic hallmark of Parkinson's disease (PD) and mutations in *SCNA* show a gene dosage effect.^1^ Therefore, intensive efforts to develop a αSyn diagnostic biomarker have been underway for decades.^2^ Because normal αSyn is ubiquitous throughout the body, biomarker assays must be specific to disease‐associated forms. Various measures of αSyn are being pursued in cerebrospinal fluid (CSF),^3^, ^4^ lacrimal/nasal/salivary secretions, gastrointestinal tract, submandibular gland (SMG), olfactory mucosa,^5^ and skin,^6^ sometimes with conflicting results.^7^ The optimal specimen(s) and methods in which to measure αSyn, weighing yield with risks of specimen acquisition, is yet to be defined.

The Systemic Synuclein Sampling Study (S4) aimed to address these knowledge gaps^8^, ^9^, ^10^ by comparing diagnostic yield of αSyn measures across CSF, saliva, blood, SMG, skin, and colon in PD versus controls. S4 initially tested assays that measure total αSyn in biofluids by ELISA or aggregated αSyn by immunohistochemistry (IHC). Results indicated that CSF and SMG αSyn were the most promising PD biomarkers, but measures of greater sensitivity, which preserve specificity, are needed.^11^ With the advent of seed amplification assays (SAA) for αSyn, we undertook this study with objectives of testing accuracy of αSyn SAA in CSF and SMG as PD biomarkers, comparing different αSyn measures in CSF and SMG, and investigating correlations with clinical severity and within‐subject relationships. We hypothesized that αSyn measured by SAA would have higher accuracy as a PD biomarker in both CSF and SMG, and that significant relationships among within‐subject measures of αSyn SAA would emerge.

## Materials and Methods

S4 was a cross‐sectional observational study that enrolled participants from October 2015 to August 2017 at six sites in the United States and Canada. Study methodology have been previously reported.^8^, ^9^, ^10^, ^11^

Institutional review board approval was obtained at each site and written informed consent was obtained from each participant.

### Study sample

As previously described,^9^ S4 aimed to recruit approximately 20 individuals with early PD, within 2 years of diagnosis and untreated for medications; 20 with moderate PD, 3–5 years from diagnosis and without motor fluctuations; and 20 with advanced PD, ≥5 years from diagnosis and with motor fluctuations and/or dyskinesias. Other inclusion criteria for the PD group included decreased striatal dopamine transporter (DAT) binding on SPECT (based on age‐matched normative data). S4 also aimed to recruit about 20 healthy controls who had normal DAT SPECT. Exclusion criteria were clinical diagnosis of dementia and presence of comorbid medical conditions that precluded specimen acquisition.

Sample characteristics of the 80 participants of the S4 cohort who contributed at least two fluid and tissue specimens (*n* = 59 PD, *n* = 21 HC) have been previously published.^11^

### Assessments and specimen acquisition

Clinical assessments in the PD and HC groups, described elsewhere in detail,^9^ included demographics, medical and neurological history and medications, Movement Disorder Society Unified PD Rating Scale (MDS‐UPDRS), and Hoehn and Yahr (H&Y) stage. Measures of non‐motor signs and symptoms included the Montreal Cognitive Assessment (MoCA), Scales for Outcomes in Parkinson's Disease‐Autonomic (SCOPA‐AUT), and University of Pennsylvania Smell Identification Test (UPSIT). Olfactory function for each subject was classified as normosmic, hyposmic, or anosmic based on normative data accounting for age and sex.^12^

DAT SPECT scan was performed and mean striatal specific binding ratio (SBR) calculated as the average of putamen and caudate SBR on right and left.

Blood, saliva, CSF, and SMG biopsy were collected and processed as described in previous reports.^8^, ^9^

### Biospecimen analysis

#### αSyn seed amplification assay (SAA) in CSF

CSF SAA was performed as previously described.^4^ Briefly, 40 μl of CSF was incubated with 1 mg/mL of purified αSyn monomers for 29 min, followed by 1 min of shaking at 500 rate per min (rpm). The buffer used in the reaction contained 100 mmol/L of PIPES (pH 6.5), 500 mmmol/L of NaCl. Protein aggregation was monitored by thioflavin T (ThT). Positive control samples containing 0.02 pg/mL of αSyn aggregates prepared in vitro were included in all experiments. Negative controls contained only buffer. Experiments were done in triplicate. Three measures of kinetics of aggregation were generated: maximum fluorescence (*F*_max_, value at which a plateau of ThT fluorescence is reached), *T*_50_ (time needed to reach 50% of maximum aggregation), and area under the curve (AUC). For each subject, a mean value across the three triplicates was calculated for each measure. Unlike total αSyn measures, CSF SAA is considered robust to hemoglobin (HGB) contamination; after confirming this in our dataset, we included in our analysis all CSF samples for which SAA results were available.

#### αSyn seed amplification assay (SAA) in SMG

SMG SAA was performed as previously described^13^ with some modifications. As the S4 specimens are formalin‐fixed paraffin embedded (FFPE) and cut onto slides, a protein‐recovery step was required. Slides were scraped with one drop ethyl alcohol (EtOH) into 1.5 mL tubes and deparaffinized (100% xylene, 100% EtOH 2×, Dry [Speed vac or open tube overnight]) followed by addition of 90 μL of Tris +0.02% SDS pH 8 buffer. To recover αSyn from the effects of formalin fixation and paraffin embedding, a kinetic assay seeding ability recovery (KASAR) method was then applied. For this purpose, a series of heating steps under the following conditions were used: 90°C for 30 min at 1500 revolutions per min (rpm), then incubation at 60°C at 1500 rpm for 90 min, and then cup sonication 90% for 4 min total (8 min – 30 sec on/30 sec off). SAA was then performed as described.^13^ In contrast to most published αSyn‐SAA assays, no further sample dilution was required in this study because the trace amount of tissue recovered from slide‐mounted biopsies was sufficiently diluted in the minimum amount of workable recovery buffer. Samples were run in quadruplicate on each plate. A fluorescence threshold was determined by averaging the baseline fluorescence (mean fluorescence of all wells measured from the first 10 readings), then adding to that the sum of 10 standard deviations of the same dataset. This results in a fluorescence threshold above which a sample is considered positive, and below which a sample is considered negative. The time at which the curve of a sample crosses the fluorescence threshold yields the “Time to Threshold” (TT) value. Another metric of SMG SAA examined was the intensity of the peak fluorescence of a sample (*F*_max_). To normalize the signals between samples, %*F*_max_ relative fluorescence units (RFU) was determined by dividing the highest mean RFU for each sample in a plate by the highest or maximal RFU for the positive control on a given plate.

#### Total αSyn measures

Total αSyn in CSF, blood, and saliva were measured using a commercially available ELISA.^8^, ^11^ Given the effect peripheral blood contamination has on total αSyn measures, specimens with hemoglobin (HGB) levels considered to be high enough to affect total αSyn measures (200 ng/mL for CSF) were excluded from analyses of total αSyn. As reported,^11^ ROC analyses identified a cutoff of ≤1800 pg/mL as optimally classifying individuals as PD versus HC (CSF ≤ 1800 is considered abnormal/positive in this analysis).

#### Aggregated αSyn in tissue

As for SMG αSyn IHC, as previously described,^10^ slides of SMG were subjected to a protease which removes non‐pathologic forms of αSyn, and then stained with the mouse monoclonal immunoglobulin 5C12 antibody. Images of stained slides were distributed to three neuropathologists blinded to diagnostic group, and each slide was rated as positive or negative for αSyn pathology. In the initial stage of S4, approximately six slides per subject were examined; subsequently, an additional 12–20 slides were examined by the same procedure, to test whether examining more slides improves sensitivity. For purposes of this analysis, a subject was considered positive for SMG αSyn if ≥1 slide(s) were rated positive for that subject.

In S4, 59 individuals with PD and 21 HC were enrolled and contributed at least two biofluids and two tissue specimens. Only those individuals who had available either CSF or SMG SAA results were included in analyses pertaining to those respective assays. Of the 59 with PD, 54 had a CSF sample available, 41 had an SMG sample available, and 38 had both CSF and SMG sample available. All 21 HC had CSF, and 14 had SMG.

### Statistical analysis

Two‐sample *t* tests were used to compare continuous variables among PD versus HC for normally distributed variables, with Wilcoxon rank‐sum tests used where nonparametric testing was appropriate. Chi‐squared test or Fisher's exact test were applied as appropriate to compare categorical variables between groups.

Sensitivity and specificity of the SAA measures in CSF and SMG as PD biomarkers (PD vs. HC) were examined using cutoff values derived from receiver operating characteristic (ROC) curves, conducted for each of the individual SAA measures (*F*_max_ and *T*_50_ for CSF, %*F*_max_ and TT for SMG). Optimal cutoffs were defined by the point on the ROC curve that yielded the highest Youden index (*J* = sensitivity + specificity−1). ROC curves were based on univariate logistic regression models and estimates of the area under the ROC curve (AUC) were also computed.

The relationships between SAA metrics and various assessments such as biofluid total αSyn measures (ELISA), as well as measures of PD disease severity were examined within the PD group using spearman rank‐order partial correlations, controlling for age.

All statistical analyses were performed using SAS version 9.4 (Cary, NC, USA).

## Results

### Sample characteristics

Sample characteristics of those with either CSF or SMG SAA results available are shown in Table 1. The PD and HC groups had similar mean age. There was a male predominance in the PD group and a female predominance in the HC group. One participant in the HC group identified as African American and one participant in the PD group identified as Hispanic or Latino; the remaining participants identified as White and not Hispanic or Latino.

**TABLE 1:** Demographics and clinical characteristics.

Variables	PD group with CSF SAA available (*n* = 54)	HC group with CSF SAA available (*n* = 21)	*P*‐value	PD group with SMG SAA available^a^ (*n* = 41)	HC group with SMG SAA available (*n* = 14)	*P*‐value
PD group (*N*)						
Early	16	NA	NA	15	NA	NA
Moderate	18			13		
Advanced	20			13		
Sex, *n* (%)						
Male	37 (68.5%)	9 (42.9%)	0.04	29 (70.7%)	6 (42.9%)	0.06
Female	17 (31.5%)	12 (57.1%)		12 (29.3%)	8 (57.1%)	
Age, years mean ± SD	63.3 ± 8.4	61.0 ± 6.3	0.25	64.4 ± 9.3	62.5 ± 6.5	0.49
Disease duration, months median (min, max)	44.9 (1.1, 245.3)	NA	NA	44.7 (2.6, 245.3)	NA	NA
MDS‐UPDRS Part I Mean ± SD	8.1 ± 5.1	2.9 ± 2.4	<0.0001	8.0 ± 5.4	2.6 ± 2.4	<0.0001
MDS‐UPDRS Part II Mean ± SD	9.4 ± 6.3	0.3 ± 0.9	<0.0001	10.0 ± 6.5	0.1 ± 0.4	<0.0001
MDS‐UPDRS Part III (OFF)^b^ mean ± SD	27.0 ± 11.6	1.1 ± 2.3	<0.0001	25.9 ± 12.1	1.1 ± 2.7	< 0.0001
Missing	5	0		1	0	
MDS‐UPDRS Total score (OFF) mean ± SD	44.7 ± 18.9	4.3 ± 3.7	<0.0001	43.7 ± 19.0	3.8 ± 4.0	<0.0001
Missing	5	0		1	0	
LEDD mean ± SD	465.6 ± 431.8	0 ± 0	<0.0001	465.9 ± 475.3	0 ± 0	<0.0001
Hoehn and Yahr (OFF)^b^, *n* (%)						
Stage 0	0 (0%)	21 (100%)	<0.0001	0 (0%)	14 (100%)	<0.0001
Stage 1	10 (20.4%)	0 (0%)		9 (22.5%)	0 (0%)	
Stage 2	33 (67.4%)	0 (0%)		26 (65%)	0 (0%)	
Stage 3	6 (12.2%)	0 (0%)		5 (12.5%)	0 (0%)	
Missing	5	0		1	0	
SCOPA‐AUT total score mean ± SD	12.7 ± 5.6	4.9 ± 3.0	<0.0001	12.2 ± 5.8	3.8 ± 2.2	<0.0001
UPSIT category, *n* (%)						
Normosmia	1 (1.9%)	13 (61.9%)	<0.0001	1 (2.4%)	8 (57.1%)	<0.0001
Hyposmia	26 (48.2%)	7 (33.3%)		19 (46.3%)	5 (35.7%)	
Anosmia	27 (50.0%)	1 (4.8%)		21 (51.2%)	1 (7.1%)	
Possible RBD present, *n* (%)	10 (18.5%)	0 (0%)	0.03	7 (17.1%)	0 (0%)	0.10
MoCA mean ± SD	27.1 ± 2.5	28.3 ± 1.2	0.007	27.1 ± 2.5	28.4 ± 1.3	0.02
Striatum SBR mean ± SD	1.1 ± 0.5	2.7 ± 0.6	<0.0001	1.2 ± 0.5	2.7 ± 0.6	<0.0001

Abbreviations: HC, healthy control; LEDD, Levodopa equivalent daily dose; MDS‐UPDRS, Movement Disorders Society Unified Parkinson's Disease Rating Scale; MoCA, Montreal Cognitive Assessment; NA, not applicable; PD, Parkinson's disease; RBD, REM sleep behavior disorder; SAA, seed amplification assay; SBR, specific binding ratio; SCOPA‐AUT, SCales for Outcomes in PArkinson's disease‐Autonomic; UPSIT, University of Pennsylvania Smell Identification Test.

^a^ SAA failed for technical reasons. One PD subject who had SMG sample available, that subject is not included in Table 1 or in the SAA results.

^b^ In five PD cases, MDS‐UPDRS III and Hoehn and Yahr OFF medication scores were not available; for these five subjects, the ON state H&Y was two in three cases (all in the moderate group), three in one case (advanced group), and zero in one case (advanced group).

### CSF αSyn SAA as a diagnostic biomarker for PD: sensitivities and specificities above 90%

Receiver operating characteristic analyses indicated that a CSF *F*_max_ cutoff of ≥1375 maximized accuracy of classification of subjects as PD versus HC. 50/54 PD were positive and 19/21 HC were negative for CSF SAA, for a sensitivity and specificity of 92.6% and 90.5%, respectively (Youden index = 83.1%). Among the PD group with negative CSF SAA, two were in the moderate PD group and two in the advanced PD group. The sensitivity of SAA was 100%, 88.9%, and 90% in the early, moderate, and advanced groups, respectively; specificity was 90.5% in all three subgroups of disease severity.

Median *F*_max_ was 3309.5 RFU in the PD group and 23 in the healthy control group. The *F*_max_ values for the four PD cases that were negative for CSF SAA were 16, 20, 146, and 481.1 RFU. Thus, one of the four cases enrolled in the PD group had an *F*_max_ value that could indicate a diagnosis multiple system atrophy (MSA).^14^

### SMG αSyn SAA in FFPE specimens as a diagnostic biomarker for PD: sensitivities and specificities above 70%

Receiver operating characteristic analyses indicated that an SMG TT cutoff of ≤26.9 h maximized accuracy of classification of subjects as PD versus HC. At that cutoff, 30/41 PD were positive and 11/14 HC were negative for SMG SAA, yielding a sensitivity and specificity of 73.2% and 78.6%, respectively (Youden Index = 51.8%). Among the 11 PD participants negative for SMG SAA, five were in the early PD group and five were in the advanced PD group.

### No relationship of αSyn SAA positivity with clinical and functional imaging measures of PD severity

Positivity of CSF SAA across subgroups of PD severity did not differ (Table 2). In addition, motor scores (MDS‐UPDRS total and part III) and DAT binding by mean striatum SBR were not significantly different in those positive versus negative for SAA measures in SMG. Examining measures of SAA as continuous variables also did not identify significant relationships to measures of disease severity (Table 3).

**Table 2:** Clinical characteristics and total α‐Synuclein values among the PD subjects with negative versus positive CSF and SMG SAA.

Variable mean (SD) or *N* (%)	PD subjects+ CSF SAA +ve (*n* = 50)	PD subjects^a^ CSF SAA −ve (*n* = 4)	PD subjects SMG SAA +ve (*n* = 30)	PD subjects SMG SAA −ve (*n* = 11)	*P*‐value (SMG SAA+ vs. SMG SAA−)
PD subgroup					
Mild	16	0	10	5	0.16
Moderate	16	2	12	1	
Severe	18	2	8	5	
Age, years	63.1 ± 8.4	66.4 ± 9.4	64.0 ± 9.8	65.4 ± 7.9	0.68
Sex, *n* (%)					
Male	34 (68.0%)	3 (75.0%)	24 (80.0%)	5 (45.4%)	0.05
Female	16 (32.0%)	1 (25.0%)	6 (20.0%)	6 (54.6%)	
UPSIT category					
Normosmic	0 (0%)	1 (25.0%)	1 (3.3%)	0 (0%)	0.48
Hyposmic	23 (46.0%)	3 (75.0%)	12 (40.0%)	7 (63.6%)	
Anosmic	27 (54.0%)	0 (0%)	17 (56.7%)	4 (36.4%)	
Disease duration, months	57.5 ± 54.8	84.9 ± 70.8	61.6 ± 63.6	59.4 ± 60.9	0.92
MDS‐UPDRS Part III (OFF)	26.9 ± 11.8	27.5 ± 10.3	27.3 ± 13.6	22.3 ± 5.8	0.11
MDS‐UPDRS total score (OFF)	44.4 ± 19.4	47.8 ± 13.3	45.0 ± 20.3	40.5 ± 15.2	0.51
LEDD	460.0 ± 443.0	535.0 ± 285.0	492.1 ± 513.4	394.5 ± 362.8	0.57
Mean striatum SBR	1.1 ± 0.5	1.0 ± 0.4	1.2 ± 0.6	1.3 ± 0.5	0.43
CSF αSyn, pg/mL					
Median (min, max)	1214.1 (667,2785.2)	1361.6 (760.1, 2018)	1320 (667, 2785.2)	1237.8 (731.8, 1799.8)	0.48
Mean ± SD	1337.6 ± 481.1	1375.3 ± 514.1	1389.9 ± 587.6	1249.1 ± 309.9	
Missing/excluded^b^	8	0	8	1	
Saliva αSyn, pg/mL					
Median (min, max)	50.6 (24, 202.8)	72.4 (24, 208.6)	59 (24, 202.8)	52.9 (24,118.4)	0.89
Mean ± SD	62.5 ± 37.6	101.7 ± 95.7	67.3 ± 41.1	69.5 ± 37.8	
Missing/excluded	10	1	6	2	
Whole blood αSyn, pg/mL × 10^7^					
Median (min, max)	2.10 (1.64, 3.79)	2.12 (1.67, 3.06)	2.16 (1.64, 3.16)	2.04 (1.64, 2.55)	0.22
Mean ± SD	2.20 ± 0.39	2.24 ± 6.05	2.24 ± 0.38	2.09 ± 0.27	
Plasma αSyn, pg/mL × 10^4^					
Median (min, max)	8.24 (0.56, 26.73)	11.42 (6.59, 11.95)	8.35 (0.65, 26.61)	10.15 (3.03, 13.09)	0.88
Mean ± SD	8.84 ± 5.81	10.35 ± 2.52	9.30 ± 5.83	9.09 ± 3.06	
Missing/excluded	0	0	0	0	
Plasma ratio (αSyn /HGB) × 10^4^					
Median (min, max)	0.31 (0.02, 1.13)	0.29 (0.16, 0.65)	0.29 (0.02, 0.78)	0.37 (0.10, 0.74)	0.53
Mean ± SD	0.34 ± 0.23	0.35 ± 0.21	0.33 ± 0.20	0.37 ± 0.19	
Missing/excluded^b^	0	0	0	0	
Serum αSyn, pg/mL					
Median (min, max)	4945.2 (1588.9, 50632.8)	8209.6 (3467, 30088.5)	4774.6 (1925.5, 50632.8)	3464.8 (1890.8, 30088.5)	0.37
Mean ± SD	8224.1 ± 8289.9	12493.7 ± 12031.3	9887.4 ± 11047.7	6331.7 ± 8129.1	
Missing/excluded^b^	0	0	1	0	
Serum ratio (αSyn /HGB)					
Median (min, max)	194.2 (58.4, 1058.4)	248.3 (144.4, 481.1)	182.3 (58.4, 1058.4)	163.1 (72.5, 481.1)	0.29
Mean ± SD	234.8 ± 176.6	280.5 ± 152.2	266.3 ± 232.5	182.2 ± 112.8	
Missing/excluded^b^	0	0	1	0	

*F*_max_, maximal fluorescence. LEDD, levodopa equivalent daily dose; MDS‐UPDRS, Movement Disorders Society Unified Parkinson's Disease Rating Scale; SAA, seed amplification assay. SBR, specific binding ratio. TT, time to threshold.

^a^ Statistical comparison not performed due to small sample size in CSF SAA‐negative group.

^b^ Specimens with hemoglobin (HGB) levels considered to be high enough to affect total αSyn measures (200 ng/mL for CSF) were excluded from analyses of total αSyn in CSF.

**Table 3:** Relationship of αSyn SAA measures to total aSyn ELISA measures in CSF, saliva, and blood, and to measures of PD disease severity.

Variables	CSF SAA: *F*_max_ Spearman's rho^a^ (*n*)^b^, *P*‐value	SMG SAA: TT Spearman's rho (*n*)^a^, *P*‐value
Measures of PD severity and age		
Age (years)	0.063 (*n* = 54), *P* = 0.651	−0.098 (*n* = 41), *P* = 0.543
Disease duration (months)	−0.062 (*n* = 54), *P* = 0.658	0.050 (*n* = 41), *P* = 0.762
MDS‐UPDRS Part III (OFF)	−0.036 (*n* = 49), *P* = 0.810	0.171 (*n* = 40), *P* = 0.299
MDS‐UPDRS total score (OFF)	0.0002 (*n* = 49), *P* = 0.999	0.160 (*n* = 40), *P* = 0.330
LEDD	−0.075 (*n* = 54), *P* = 0.594	0.102 (*n* = 41), *P* = 0.533
Mean striatum SBR	−0.211 (*n* = 54), *P* = 0.129	−0.182 (*n* = 41), *P* = 0.262
Total αSyn measures (ELISA)		
CSF αSyn, pg/mL	**−0.311 (*n* = 54), *P* = 0.024**	−0.004 (*n* = 38), *P* = 0.983
Saliva αSyn, pg/mL	−0.079 (*n* = 50), *P* = 0.591	−0.069 (*n* = 38), *P* = 0.684
Whole Blood αSyn, pg/mL × 10^7^	−0.232 (*n* = 54), *P* = 0.094	0.196 (*n* = 41), *P* = 0.227
Plasma αSyn, pg/mL × 10^4^	**−0.289 (*n* = 54), *P* = 0.036**	−0.027 (*n* = 41), *P* = 0.868
Serum αSyn, pg/mL	−0.143 (*n* = 54), *P* = 0.307	0.263 (*n* = 41), *P* = 0.101

*P* < 0.05 are in bold. *F*_max_, maximal fluorescence. LEDD, levodopa equivalent daily dose; MDS‐UPDRS, Movement Disorders Society Unified Parkinson's Disease Rating Scale; SAA, seed amplification assay; SBR, specific binding ratio; TT, time to threshold.

^a^ Spearman rank‐order partial correlations adjusting for age.

^b^ *n* for which specified correlation coefficient was generated.

### Relationship of αSyn SAA to ELISA and IHC measures

The relationship between SAA in CSF and SMG and total αSyn (ELISA) measures across all biofluids is shown in Table 3. There was a moderate inverse correlation between CSF SAA *F*_max_ and total αSyn in both CSF (Spearman's ρ = −0.31; *P* = 0.024) and plasma (ρ = −0.29; *P* = 0.04), indicating that higher *F*_max_ correlated with lower total CSF and plasma αSyn.

**FIGURE 1:**
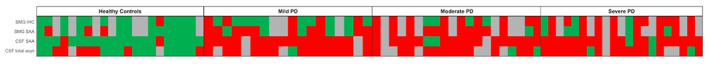
Qualitative heat map depicting results of aSyn using different measures: CSF and SMG SAA, total aSyn by ELISA, and aggregated aSyn by IHC. Each column represents one subject. Red indicates a positive assay, green a negative assay, and gray no data available (assay result not available and/or, for CSF total aSyn, hemoglobin >200 ng/mL).

All subjects enrolled in S4 who contributed at least two tissues and two fluids (*n* = 80) are included in the following analyses which contrast the different measures of αSyn in the overall cohort. The prevalence of positivity for each of four measures of αSyn is depicted in Figure 1. Among PD subjects with results available for CSF and SMG SAA, 25/38 (65.8%) were positive for both. The individual enrolled in the PD group who had negative CSF SAA but whose *F*_max_ value was 481 RFU (a value that could indicate MSA), had a positive SMG SAA.

In considering CSF αSyn measures, 58/59 PD subjects enrolled in S4 had at least one measure positive. Among the *n* = 42 in the PD group who were positive for CSF SAA, CSF total αSyn was positive in 37 (88.1%). Notably, three samples were positive for CSF total αSyn but not for CSF SAA. In only one of the PD cases with both assays available were results negative on both tests. Among the HC, one was positive for both CSF SAA and total αSyn.

In contrasting SMG SAA to IHC among the PD group, of the 41 with both results available, 20 (48.8%) were positive for both. Among the 30 subjects positive for SMG SAA, SMG IHC was positive in 20 (66.7%). Among 11 participants negative for SAA, IHC was positive in four participants. Among the 17 PD participants negative for IHC, SAA was positive in 10 participants. Notably, three HC were positive for SMG SAA, but were negative for SMG IHC, whereas the one SMG IHC positive HC was negative for SMG SAA.

In the PD cohort, 12/59 (20.34%) were positive for all four αSyn measures compared here. Importantly, only one PD subject was negative across all available measures (though missing data for this subject limit interpretation of this finding). Of 35 participants who had either SMG measure positive, all also had a CSF measure positive. Of those with CSF positive by either measure who also had both SMG measures available, 12/26 (46.2%) had positive SMG by either measure.

Two controls were positive in both CSF and SMG: one was IHC positive in SMG and had positive CSF SAA, whereas the other had SMG SAA positive and CSF total αSyn positive.

Comparing total αSyn measures in CSF and blood in relation to SAA positivity in CSF or SMG in the PD group revealed no significant differences (Table 2).

## Discussion

In this study, αSyn SAA, which has high specificity for disease‐associated αSyn, yielded high accuracy as a PD diagnostic biomarker in CSF, a central source, and moderate accuracy in SMG, a peripheral tissue proximal to the CNS. These findings are superior to the performance of previously examined measures,^11^ where total αSyn quantification in CSF showed high sensitivity (87%), but low specificity (63%); and IHC in SMG for aggregated αSyn had high specificity (92.9%) but low sensitivity (56.1%). The improved specificity of αSyn SAA also accounts for the greater within‐subject concordance among αSyn measures that we demonstrate here, as compared to the initial S4 results.^11^

Biomarkers are critically needed to facilitate diagnosis of PD, especially early on in the disease course where diagnostic accuracy may be low, particularly among older indivdiuals.^15^ The advent of SAAs brings the opportunity to detect the underlying proteinopathy in an individual with parkinsonism^16^; αSyn SAA thus provides complimentary information to dopamine transporter (DAT) imaging, which detects dysfunction/degeneration of the nigrostriatal system, but is agnostic to the underlying etiology. In S4, we found that CSF SAA was sensitive and specific for PD diagnosis, even in early stages of the disease, as reported in several other studies.^3^, ^17^ As a PD biomarker, CSF SAA outperformed CSF total αSyn, yielding higher sensitivity (93% vs. 87%) and dramatically improved specificity (90% vs 63%). This is not surprising considering that total αSyn measures likely reflect a combination of various forms of both normal and pathologic αSyn.^18^ αSyn is a 140 amino acid protein ubiquitous throughout the body. In PD, it misfolds and aggregates in a multistep process whereby monomeric αSyn nucleates into soluble oligomers and then into large insoluble fibrils.^19^ The assay we used to quantify total αSyn in the S4 fluids recognizes only residues 1–130 of αSyn.^20^ Total αSyn levels in CSF are lower in PD compared to HC, though there is substantial overlap in values between groups.^21^ In other neurodegenerative diseases such as Alzheimer's disease (AD), CSF levels of αSyn are elevated, which has been postulated to result from release of intracellular αSyn into the extracellular space due to neuronal degeneration.^18^ Further complicating the interpretation of total αSyn values is that while the full‐length protein is the most common form in the human body, there are at least three isoforms produced by alternative splicing. αSyn also undergoes several post‐translational modifications (PTMs), some of which are disease‐specific,^19^ but are not measured by the assay applied to S4 specimens.^22^ Another factor hampering the comparability of immunoassays are the antibodies used and the respective binding sites that leads to variability.^18^, ^22^ As different assays for αSyn are being developed, relating them to total αSyn values remains important, especially since ratios of different measures of the protein may improve diagnostic accuracy.^18^, ^23^ The exact mechanism behind SAA is not yet fully understood, but the high specificity of SAA lies in its leveraging of the ability of disease‐associated αSyn to induce spontaneous aggregation of nonfibrillar αSyn. Thus, it is protein and disease specific. In addition to the greater specificity for seed‐competent αSyn, CSF SAA is not sensitive to contamination by RBCs.^24^, ^25^

In line with the above, in examining the relationship between CSF αSyn measured by SAA and ELISA, we found a moderate but significant relationship between CSF *F*_max_ and total αSyn. While the relationship is conceptually intuitive, any significant relationship between the two measures would be expected to be of small effect size, given the aforementioned considerations related to measures of total αSyn by antibodies, whereby only a small percentage of the overall measure likely reflects pathologic forms of αSyn.^22^ In addition, the SAA does not quantitate the absolute amount of pathologic αSyn, and we cannot exclude the possibility that there are pathologic components of total αSyn not amplified by the SAA. Our findings are, however, in contrast to Kang *et al* ^26^ as well as Poggiolini *et al*,^3^ where even in a larger number of samples, a significant relationship between CSF αSyn and *F*_max_ and/or *T*_50_ was not found. Given that the S4 protocol and the study of Kang *et al* ^26^ followed similar SOPs for CSF collection, preanalytical factors are unlikely to account for these discrepant findings.

Our data indicate that the two approaches to measuring αSyn in CSF (SAA and ELISA) provide complementary information. Even with the high sensitivity of CSF SAA, there were three PD subjects negative for CSF SAA but positive for total αSyn by ELISA. One of these subjects had an *F*_max_ value that was significantly higher than normal controls but lower than in PD. This pattern has been described in individuals with MSA^3^, ^14^; thus, this patient may have been misdiagnosed as having PD. As for specificity, the SAA measures for CSF αSyn did improve specificity when compared with the quantification of total αSyn. We cannot exclude the possibility that the one HC who was positive for CSF SAA and total αSyn, or those positive for both SMG and CSF αSyn, are prodromal for PD or other αSyn‐mediated diseases; S4 did not collect longitudinal data and we are unfortunately not able to assess this further.

Investigating the distribution of αSyn in peripheral tissue informs proposed models of spread of αSyn during the pathophysiological cascade that are alternately posited to occur first in the CNS or first in the PNS, the latter in the gastrointestinal tract and elsewhere. Chahine *et al* ^11^ and Visanji *et al* ^27^ were not able to consistently detect pathologic αSyn in the distal colon in PD, but more proximal regions of the GI tract may harbor higher concentrations of specific forms of pathologic αSyn. The SMG is a structure of particular interest in studying pathologic αSyn in the GI tract; salivary glands are in the most rostral regions of the GI tract, and a rostro‐caudal gradient of pathologic αSyn has been postulated in PD.^28^ In a study of SMG SAA in postmortem tissue from 13 PD and 16 controls, the sensitivity of SMG SAA for PD was 100% and specificity was 94% in frozen specimens and 76% sensitive and 100% specific in FFPE specimens.^13^ It is important to consider that SAA on FFPE specimens is challenging due to the need to remove the paraffin from thin SMG sections and possible conformational changes or cross‐linking induced by heat and formalin fixation; greater sensitivity and specificity of SMG SAA has been obtained in frozen specimens.^13^ In contrasting SMG SAA and IHC, the expertise and logistics required to interpret IHC^8^ may make SAA a more appealing assay to study pathologic αSyn in SMG and other peripheral tissues in the future, although the results from S4 and other IHC studies indicate that IHC offers additional spatial and quantitative information as compared with SAA and thus may remain of utility, particularly if sensitivity can be improved.

As for the relationship of central (CSF) and peripheral (SMG) measures of αSyn, not surprisingly, there were not any instances of PD subjects having positive SMG but not positive CSF. While it has been hypothesized that PD pathology may begin outside the CNS,^29^ human autopsies indicate that peripheral αSyn pathology in the absence of CNS synuclein pathology is extremely rare in established disease.^30^ S4 did not enroll prodromal cases and thus the temporal relationship of peripheral versus central αSyn measures cannot be examined with our data. Importantly, even with missing data for some measures due to inadequate tissue or elevated CSF Hb, only one PD subject was negative across all available measures. As for the metric of SAA in tissue versus CSF, whereby in CSF *F*_max_ had optimal accuracy, in SMG, the TT metric was optimal and *F*_max_ less sensitive. This may have been due to heat, xylene and EtOH used to remove paraffin, and/or formalin‐induced cross‐linking of αSyn aggregate seeds, the dilute nature of the processed FFPE samples, variability in the total amount of target tissue harvested from each slide, or other factors yet to be investigated.

A lack of relationship between αSyn SAA measures and clinical measures of PD disease severity is consistent with other studies,^3^, ^26^ indicating that the measures of SAA applied in this study are not of utility as objective biomarkers of PD severity. Several promising methods are under development that employ adaptations of the SAA as measures of disease severity. One approach involves end point dilution. Addition of different known concentrations of aSyn seeds into CSF from healthy individuals or dilutions of PD CSF samples have shown that the assay is able to differentiate samples with distinct quantities of aggregates.^31^ Indeed, higher quantities of seeds lead to a faster kinetic of aggregation. In another, multiplex approach, ELISA was applied to the end products of SAA to quantitate disease‐specific oligomers^32^; levels of CSF‐seeded αSyn oligomers had moderate, significant correlations with UPDRS and Hoehn and Yahr stage.

S4 study limitations include the small sample size in PD subgroups, limiting comparisons. In addition, the cohort lacks racial and ethnic diversity, limiting generalizability. Nevertheless, S4 remains unique and valuable with the various intraindividual tissue and fluid collection in subjects with different PD stages and HC.

In conclusion, the S4 study, by comparing αSyn measured by SAA and ELISA in CSF and SAA and IHC in SMG, illustrates the higher sensitivity and specificity of SAA in these matrices. Our results support the value of other αSyn measures to inform further development of αSyn as a PD biomarker and indicate that multimodal and multifocal assessment of αSyn biomarkers is informative.

## Conflict of Interest

The authors declare no conflicts of interest related to this work.
